# Insights into lysosome-related organelle biogenesis: melanosome as a model organelle

**DOI:** 10.3389/fcell.2025.1758081

**Published:** 2026-01-12

**Authors:** Duarte C. Barral, Cédric Delevoye, Lionel Larue, Miguel C. Seabra, Graça Raposo, Subba Rao Gangi Setty

**Affiliations:** 1 iNOVA4Health, NOVA Medical School, Faculdade de Ciências Médicas, NMS, FCM, Universidade NOVA de Lisboa, Lisboa, Portugal; 2 Université Paris Cité, INSERM UMR-S1151, CNRS UMR-S8253, Institut Necker Enfants Malades, Paris, France; 3 INSERM U1021, Normal and Pathological Development of Melanocytes, Institut Curie, PSL Research University, Orsay, France; 4 Centre National de la Recherche Scientifique (CNRS) UMR 3347, University Paris-Sud, University Paris-Saclay, Orsay, France; 5 Institut Curie, PSL Research University, Sorbonne University, CNRS, UMR144, Cell Biology and Cancer, Paris, France; 6 Department of Microbiology and Cell Biology, Indian Institute of Science, Bangalore, India

**Keywords:** keratinocyte, lysosome-related organelle, melanocyte, melanin, melanosome biogenesis, retinal pigment epithelium, skin pigmentation and photoprotection

## Abstract

Lysosome-related organelles (LROs) encompass specialized intracellular compartments that share features with lysosomes while fulfilling distinct physiological roles, with melanosomes representing the best-studied example. Melanosome biogenesis relies on coordinated trafficking, sorting, and membrane remodeling mechanisms that diverge from the canonical endolysosomal pathways. These organelles ultimately serve as the primary sites of melanin synthesis and deposition. In the skin, melanin is produced by melanocytes and transferred to keratinocytes, where it achieves its essential photoprotective role. Melanin is a remarkably diverse and ancient polymer, with eumelanin, pheomelanin, and neuromelanin constituting the major mammalian forms. Understanding melanin biology also requires tracing the origins of melanocytes, which were once thought to derive exclusively from the neural crest but are now known to arise from multiple embryonic lineages. This expanded view of melanocyte ontogeny has revealed unexpected pigment cell populations in several internal organs. Beyond these developmental aspects, melanin performs multifaceted physiological functions that extend far beyond photoprotection of the skin. Here, we discuss the current knowledge on the origin of melanosomes from endosomal precursors, the transfer of melanin from melanocytes to keratinocytes, and its fate in these recipient cells within the epidermis. Additionally, the intriguing mysteries surrounding melanosomes in the retinal pigment epithelium are addressed, as well as the broader diversity, origins, and physiological roles of melanin in other cell types. Taken together, these perspectives highlight the melanosome as both a model LRO and an organellar hub for deciphering melanin diversity, cellular origins, and the wide-ranging physiological roles of this pigment in vertebrate biology.

## From endosomes to pigment organelles: building the melanosome

Lysosome-related organelles (LROs) are cell type–specific, membrane-bound compartments that share common features with lysosomes but fulfil specialized functions. They contain lysosomal membrane proteins and hydrolases, often maintain an acidic pH, and can be accessed by endocytic tracers (Marks et al., 2013; Delevoye et al., 2019). Among LROs, melanosomes are the best characterized and serve as a prototypical model for LRO biogenesis (Le et al., 2021). Melanosome maturation proceeds through four morphologically distinct stages, reflecting their functional differentiation. Stage I melanosomes correspond to early/sorting endosomes containing intraluminal vesicles that initiate fibril formation (Hurbain et al., 2008; van Niel et al., 2015). PMEL (also known as gp100 or silver) is structurally related to the amyloid precursor protein (APP) and is processed within stage I melanosomes forming the intraluminal fibrillar matrix of stage II ellipsoid premelanosomes (Watt et al., 2013; Ma et al., 2025). These fibrils provide the scaffold for melanin deposition (Bissig et al., 2016) and are proposed to detoxify reactive intermediates produced during melanogenesis. Active melanogenesis begins in stage III melanosomes, to which melanin-synthesizing enzymes—tyrosinase (TYR), tyrosinase-related protein 1 (TYRP1), and dopachrome tautomerase (DCT, aka TYRP2)—as well as other cargoes are specifically delivered (Bowman et al., 2019; Le et al., 2021). Finally, stage IV melanosomes are fully melanized, masking their internal structure. The formation of melanosomes diverges from the conventional endolysosomal pathway, reflecting their specialized function (Raposo et al., 2001). Over the past 25 years, research from several groups has provided an integrated view of the cellular and molecular mechanisms driving melanosome biogenesis (Bowman et al., 2019; Le et al., 2021; Salavessa et al., 2025).

Insights into melanosome biogenesis and cargo delivery have greatly benefited from studies of genetic disorders affecting pigmentation. Oculocutaneous albinism (OCA) and ocular albinism (OA) are characterized by hypopigmentation of the skin, hair, and eyes (Fernandez et al., 2021). In non-syndromic albinism, mutations occur in melanocyte-specific genes such as *TYR*, *GPR143/OA1*, *OCA2*, *OCA3*, and *OCA7*. In syndromic forms, such as Griscelli and Hermansky–Pudlak syndromes (GS and HPS), the mutated proteins are involved in melanosome transport or biogenesis, respectively (Bowman et al., 2019). Studies on HPS proteins that are ubiquitously expressed, including the adaptor complex AP-3 and the biogenesis of lysosome-related organelles complexes (BLOC-1, BLOC-2, BLOC-3), have shed light on the trafficking steps and dynamic organization underlying melanosome formation and distinction from canonical lysosomes. Of interest, melanocytes exploit ubiquitous trafficking machineries and adapt and remodel their endo-secretory organelles and derived membrane transport intermediates to generate melanosomes.

In the endocytic pathway, early/sorting endosomes serve as platforms for PMEL processing. The luminal domain of PMEL is sorted into intraluminal vesicles through interaction with the tetraspanin CD63, whereas its transmembrane domain is directed to lysosomes for degradation via an ESCRT-dependent mechanism (van Niel et al., 2011). Whether these organelles represent specialized subpopulations of early endosomes or transient sorting intermediates remains unclear. Among regulators of early melanosome maturation, OCA7 controls melanosomal pH and PMEL processing (Beyers et al., 2022). Proteins mutated in HPS are central to trafficking and organelle dynamics, required for melanin synthesis. AP-3, BLOC-1, and BLOC-2 localize to tubular recycling endosomes (REs) (Di Pietro et al., 2006; Setty et al., 2007). While REs typically recycle cargoes to the plasma membrane, in melanocytes, a subset establishes close contacts with melanosomes (Delevoye et al., 2009; Dennis et al., 2015). AP-3 mediates sorting of TYR, whereas AP-1 sorts both TYR and TYRP1 (Theos et al., 2005). AP-1 interacts with the kinesin motor KIF13A, forming a tripartite complex with TYRP1 (Delevoye et al., 2009). KIF13A also interacts with the BLOC-1 complex, which promotes curvature of negatively charged (*e.g*., phosphatidylinositol-4-phosphate (PI4P)-positive) membranes to drive recycling endosomal tubule formation from sorting endosomes (Jani et al., 2022; Zhu et al., 2022), a process facilitated by BLOC-1-mediated coordination of both the microtubule and actin cytoskeletons (Delevoye et al., 2016). Small GTPases play essential roles in regulating trafficking toward melanosomes. Rab32 and Rab38 recruit BLOC-2, AP-1, and AP-3 to direct cargo delivery to melanosomes (Bultema and Di Pietro, 2013). Rab22a promotes assembly of a BLOC-1–BLOC-2–KIF13A complex on early/sorting endosomes to generate REs (Shakya et al., 2018). In parallel, TYRP2/DCT and MART-1 are transported to maturing melanosomes via diversion of the secretory pathway in a Rab6- and ELKS-dependent manner (Patwardhan et al., 2017).

Melanosome maturation requires tight regulation of organelle size, pH, and molecular composition. For example, the two-pore channel TPC2 controls melanosomal pH and size by mediating calcium release (Ambrosio et al., 2016). Recycling of cargo from melanosomes is also required to maintain their size and composition, which can be carried out by tubular membrane intermediates whose fission depends on myosin VI and actin polymerizing machineries (Ripoll et al., 2018). In addition, Rab38 and its guanine exchange factor BLOC-3 regulate melanosomal tubular carrier formation and the recycling of the N-ethylmaleimide-sensitive factor (NSF) attachment protein receptor (SNARE) VAMP7 (Dennis et al., 2016). Altogether, these studies and many others not referenced here have illuminated the complex biogenesis of melanosomes, the roles of pigment-related disease gene products, and the interplay between membrane trafficking and organelle dynamics (Salavessa et al., 2025). Nevertheless, many questions remain regarding the structural and biochemical coordination between trafficking complexes and associated membrane functions. Future investigations will likely extend this knowledge to other LROs, advancing our understanding of specialized organelle formation and function.

## From melanin transfer to genome photoprotection: melanin processing in keratinocytes

Skin pigmentation and, hence, photoprotection rely on the crosstalk between melanocytes—the pigment producing cells—and keratinocytes—the pigment acceptors. Pigmented melanosomes accumulate in melanocyte dendrites through anterograde trafficking along microtubules, regulated by the complex formed by Rab1a, kinesin-1/(Kif5b + KLC2) and kinesin-interacting protein (SKIP) (Ishida et al., 2012; Ishida et al., 2015). Once at the cell periphery, melanosomes in skin melanocytes are tethered to the actin cytoskeleton via a complex formed by Rab27a, melanophilin and myosin Va, whose mutations cause GS, as well as through the interaction between Rab27a and synaptotagmin-like protein (Slp)-2a (Fukuda et al., 2002; Strom et al., 2002; Kuroda and Fukuda, 2004; Hume et al., 2007).

Four different models have been proposed to explain the mechanism of melanin transfer (Bento-Lopes et al., 2023; Bao et al., 2025). Overwhelming evidence coming from organotypic models that mimic human skin structure and human skin biopsies favors the exo/phagocytosis transfer route. In this model, pigmented melanosomes fuse with the plasma membrane (PM) of melanocytes, releasing melanocores (*i.e.*, the intraluminal melanin core of a melanosome devoid of its limiting membrane) to the intercellular space, which are subsequently phagocytosed by keratinocytes. This implies that melanocores essentially lack melanosomal membrane components like TYRP1 (Tarafder et al., 2014; Benito-Martínez et al., 2025) and acquire a new membrane derived from the PM of keratinocytes upon internalization. Several regulators of melanosome exocytosis, and hence melanocore secretion and transfer, have been described. These include Rab11b and the exocyst complex subunits Exo70 and Sec8 (Tarafder et al., 2014; Moreiras et al., 2020), which are involved in melanosome exocytosis under basal conditions. In contrast, Rab3a regulates this process upon stimulation with soluble factors present in keratinocyte-conditioned medium (Cabaco et al., 2022). Since several SNAREs, namely, vesicle-associated membrane protein (VAMP)-2, soluble NSF attachment protein (SNAP)-25, SNAP-23, syntaxin-4 and α-SNAP (Araki et al., 2000; Scott and Zhao, 2001), were detected fractionating with melanosomes alongside Rab3a, it is possible that these are involved in the fusion of melanosomes with the PM of melanocytes.

While not considered professional phagocytes, keratinocytes possess phagocytic capacity. Consistently, melanocores can be internalized by keratinocytes through phagocytosis in a manner dependent on the Rho small GTPases Rac1 and Cdc42, but not RhoA (Correia et al., 2018; Moreiras et al., 2022). Additionally, toll-like receptor 3 (TLR3) activation can enhance melanin internalization by recruiting RhoA and Cdc42 (Koike et al., 2019). Furthermore, the activation of the G-protein-coupled receptor (GPCR) protease-activated receptor-2 (PAR-2) stimulates keratinocyte phagocytic activity (Sharlow et al., 2000). Nevertheless, a key unanswered question remains: the identity of the keratinocyte surface receptor(s) with which melanocores can interact. Once transferred to keratinocytes, melanocores are stored in specialized compartments that differ from classical lysosomes and protect nuclear DNA from ultraviolet (UV) radiation. These compartments are melanocore-positive but differ from melanosomes in their membrane composition and were proposed to be named melanokerasomes (MKSs). While these specialized organelles need to have their own designation, the term MKS remains a suggestion that the pigment cell community should validate. MKSs share molecular features with late endosomes and lysosomes, such as the presence of LAMP1 and CD63, yet exhibit a reduced degradative capacity and acidity (Correia et al., 2018; Hurbain et al., 2018; Benito-Martínez et al., 2025; Neto et al., 2025). This adaptation likely ensures that melanin can persist long enough within keratinocytes to fulfil its photoprotective role (Salavessa et al., 2025).

Although the precise steps of MKS formation remain to be defined, they seem to follow the conventional endocytic pathway before shifting to long-lived, non-degradative storage lysosomes (Correia et al., 2018; Benito-Martínez et al., 2025; Neto et al., 2025). This maturation process likely involves membrane trafficking regulators such as Rab GTPases (Marubashi and Fukuda, 2020), whose exact contributions are only beginning to emerge (Neto et al., 2025). Rab7 was shown to be required for the fusion of MKSs with degradative lysosomes, prior to their conversion into a melanin storage compartment (Neto et al., 2025). In parallel, autophagy appears to influence MKS homeostasis, as treatment with autophagy modulators alters pigmentation in keratinocytes and skin explants (Murase et al., 2013; Kim et al., 2020). Upon maturation, MKSs organize in a supranuclear “umbrella” that concentrates pigment above the nuclei of keratinocytes, forming the so-called melanin cap or microparasol (Kobayashi et al., 1998; Gibbs et al., 2000). This spatial organization is thought to be a critical determinant of photoprotection (Del Bino et al., 2006; Brenner and Hearing, 2008). The formation of supranuclear melanin caps relies on the concerted action of at least two major cytoskeletal systems: microtubules enabling long-range retrograde transport toward the perinuclear region (Byers et al., 2003; Byers et al., 2007; Neto et al., 2025), and keratin (KRT) intermediate filaments (KRT5/14), which were recently shown to mechanically maintain MKSs close and atop the nucleus through cage-like cytoskeletal structures (Benito-Martínez et al., 2025). These systems cooperate through cytolinkers, like plectin, which bridges microtubules and intermediate filaments to vertically position the melanin cap (Benito-Martínez et al., 2025) — a process disrupted when microtubule polymerization or F-actin dynamics are impaired in skin explants (Castellano-Pellicena et al., 2021). Together, this suggests that keratinocytes coordinate their cytoskeletal networks to create and maintain a three-dimensional pigment shield that reduces the mutagenic effects of UV exposure.

The functional importance of this 3D-pigmented microparasol becomes evident in genetic skin disorders affecting the cytoskeleton. Interestingly, mutations in *KRT5* or *KRT14*, which underlie Dowling-Degos disease, epidermolysis bullosa simplex, and other keratinopathies, not only cause skin fragility but also lead to pigmentation abnormalities (Betz et al., 2006; Salavessa et al., 2025). Moreover, patients often display hyperpigmented lesions, yet melanin appears dispersed rather than concentrated above the nuclei of keratinocytes. Such dispersion compromises photoprotection *in vitro*, despite the presence of abundant pigment (Benito-Martínez et al., 2025), which may contribute to an increased risk of skin cancer. Intriguingly, evidence of the existence of tethers between MKSs and the nuclear envelope, which may play a role in maintaining MKS positioning, was recently reported (Neto et al., 2025). These insights highlight how pigment biology in keratinocytes extends far beyond passive storage. Instead, it represents a dynamic system of organelle biogenesis, trafficking, and cytoskeletal dynamics that has evolved to maximize genome photoprotection (Salavessa et al., 2025). Future research should clarify how these processes vary across skin phototypes (Szabo et al., 1969; Minwalla et al., 2001; Thong et al., 2003; Yoshida et al., 2007; Hurbain et al., 2018), how they become altered in disease, and whether they can be modulated therapeutically for the treatment of pigmentary disorders.

## The mysteries of melanosomes in the retinal pigment epithelium

Unlike the maturation, transport and exocytosis of melanosomes in skin melanocytes, much less is known about melanosomes of the retinal pigment epithelium (RPE). RPE melanosomes exhibit several important differences from those of skin melanocytes, which have been well established. Melanosomes reside within the RPE for as long as the organism survives, as these cells retain the pigment (unlike neighbouring choroidal melanocytes, which continue to produce pigment throughout life but at a slower rate). Melanogenesis occurs early in the development of RPE and then stops, either completely or partially, as low levels of melanin synthesis have been described during adulthood (Schraermeyer, 1993). The microphtalmia-associated transcription factor (MITF) appears to be the main driver of melanogenesis, promoting the expression of enzymes necessary for melanin synthesis, as observed in skin melanocytes. The rationale for the loss of melanogenesis in the RPE appears straightforward. The number of melanosomes produced during the embryonic period enables the epithelium to be loaded with a functionally significant amount of melanin, unlike melanocytes, whose job is to produce a maximum of melanin to be transferred. By birth, RPE cells fully differentiate and become post-mitotic. At this time, the main function of RPE is to support adjacent photoreceptors by turning over photoreceptor outer segments (POS), regulating the visual cycle, and sustaining them metabolically, thereby ensuring adequate glucose levels.

One obvious function of RPE melanosomes is to absorb light scattering in the back of the eye and therefore contribute to better focus in vision, as photoreceptors receive more direct light and less scattered light. In some vertebrate species, namely, fish and amphibians, RPE melanosomes are highly motile in response to light, thereby controlling the amount of light that reaches the photoreceptors. Beyond that, the functional relevance of RPE melanin remains largely unknown, despite some evidence suggesting that it may have protective functions in response to stress and help maintain homeostasis. Some other functions ascribed to RPE melanosomes include serving as anti-oxidative agents through the scavenging properties of melanin and thermal regulation as light absorption dissipates heat (Burke et al., 2011). Additionally, RPE melanosomes share some characteristics with lysosomes, including the presence of luminal lysosomal hydrolases, LAMP1 at the membrane, and phagocytosed POS (Schraermeyer et al., 1999). The localization of POS to RPE melanosomes could be due to melanosome-phagosome fusion; however, whether this is a common occurrence or an exception under normal conditions is still unknown (Schraermeyer et al., 1999). Given the lifelong load of phagocytosis of POS by the RPE and the accumulation of POS-dependent lipofuscin over time, the ability of melanosomes to assist lysosomes in degrading ingested material becomes crucial. Interestingly, a new organelle appears in the RPE with age, called melanolipofuscin, which combines melanin and lipofuscin in one compartment (Boulton, 2014). The significance and/or function of this unique organelle needs to be further explored.

If indeed melanosomes aid lysosomes, then melanin/melanosomes could be protective against RPE degeneration and death. Longitudinal studies have demonstrated a gradual loss of RPE melanosomes with age (Feeney-Burns et al., 1984; Pollreisz et al., 2018). Since there is no significant *de novo* melanogenesis in adult RPE as discussed above, this decrease with age can lead to an increased susceptibility to age-related degenerative diseases of the eye.

Age-related macular degeneration (AMD) is a common age-related disease associated with vision loss, and the degeneration of RPE appears to play a major role. Caucasians are 5-fold more susceptible to AMD than those of African descent (Klein et al., 2006). A retrospective analysis regarding the incidence of AMD in patients who were prescribed L-DOPA (a precursor of melanin synthesis) revealed they were less likely to develop AMD, and in those who did develop the disease, the age of onset was significantly delayed (Brilliant et al., 2016). Also, macular pigment optical density (MPOD), a measurement of the level of pigment in the RPE, is reduced in patients with AMD. Interestingly, MPOD is age-dependent in AMD patients but not in healthy controls, suggesting a gradual loss of pigment during the development of AMD (Kaya et al., 2012). Whether this decrease in melanosomes with age and disease can be reverted or upregulated as a novel therapeutic approach remains to be shown. In summary, it is fascinating that the melanosome, generated by the same regulatory pathways and containing the same essential components in both skin melanocytes and RPE, behaves in such disparate ways in both cell types.

## Melanin: diversity, origins, and physiological functions

Melanin is a complex and evolutionary ancient polymer found in a wide range of living organisms. At least five types of melanin have been identified: eumelanin, pheomelanin, neuromelanin, allomelanin, and pyomelanin. Among these, allomelanin is produced exclusively by plants and fungi, while pyomelanin is found in certain bacteria. In mammals, three main forms are relevant: eumelanin (brown-black) and pheomelanin (yellow-red), both synthesized by melanocytes, and neuromelanin, a pigment similar to eumelanin, which is present in specific regions of the brain, such as the *substantia nigra* and the *locus coeruleus*.

Until recently, melanocytes were thought to originate solely from the neural crest and to localize mainly in the epidermis, with a few exceptions, such as the eye and inner ear. Additionally, TYR was long thought to be specific to melanocytes. However, this view is now changing, with recent studies showing that melanocytes can also derive from early ectodermal cells (Kinsler and Larue, 2018) and Schwann cell precursors (SCPs) (Adameyko et al., 2009). The fate of SCPs—toward melanocytes or Schwann cells—is determined by the balance between forkhead box D3 (FOXD3) and MITF transcription factors, regulated in turn by Wnt/β-catenin signaling (Colombo et al., 2022). SCP-derived melanocytes are predominantly located in the ventral regions of the limbs (Colombo et al., 2022). These findings suggest that melanocytes have at least three embryonic origins, with the neural crest remaining the principal source. Interestingly, melanocytes have also been identified in several internal organs, including the olfactory bulb in the brain (Gudjohnsen et al., 2015) and the heart—particularly in the ductus arteriosus and cardiac valves (Brito and Kos, 2008; Yajima and Larue, 2008; Colombo et al., 2011). Notably, activation of the Wnt/β-catenin signaling pathway during cell fate specification promotes melanocyte differentiation at the expense of the smooth muscle lineage in the ductus arteriosus, leading to its failure to close at birth and resulting in a patent *ductus arteriosus* (Yajima et al., 2013). In addition, introducing an oncogenic Braf mutation in melanoblasts leads to abnormal proliferation, resulting in severe cardiac defects and neonatal lethality in mouse models (Dhomen et al., 2010).

While melanin’s most recognized function is skin and visual protection against UV radiation, its roles in internal organs are less well understood. One hypothesis suggests that melanin’s ancient molecular structure enables it to act as a protective buffer against various stressors, including UV radiation, oxidative damage, heavy metals, ionizing radiation, and toxins. For instance, neuromelanin may offer neuroprotective effects by sequestering potentially harmful metals such as iron and copper. The loss of neuromelanin has been associated with neurodegenerative diseases such as Parkinson’s disease (PD), characterized by the progressive depigmentation of the substantia nigra. In the heart and cardiac valves, melanocyte-like cells have been discovered in the sinoatrial node, the heart’s natural pacemaker (Levin et al., 2009). Interestingly, melanocyte pigmentation stiffens murine cardiac tricuspid valve leaflet (Balani et al., 2009) and melanocytes contribute to elastogenesis in the developing murine aortic valve (Nasim et al., 2024). In the inner ear, melanocytes are found within the cochlear *stria vascularis*, where they help maintain the ionic composition of the endolymph—crucial for auditory transduction. They are essential for preserving high potassium concentrations and the endocochlear potential necessary for hair cell function. Melanin also appears to provide protection against oxidative stress and heavy metal toxicity, which may play a role in preserving hearing, particularly during aging or after noise exposure (Coutant et al., 2024). Melanocytes are also present in the olfactory bulb and may serve neuroprotective functions by acting as antioxidants in a metabolically active region (Gudjohnsen et al., 2015). They might also modulate local inflammation and act as a chemical barrier between olfactory neurons and the central nervous system, akin to glial support cells. Although their precise role in olfaction remains to be clarified, melanocytes may be involved in conditions affecting smell, including neurodegenerative disorders such as PD. In summary, melanin is an ancient, multifunctional pigment produced in diverse tissues and organisms, with melanocytes arising from multiple embryonic sources and contributing to functions far beyond UV protection. In addition to the roles in the skin, melanin and melanocytes support cardiac, auditory, visual, and potentially olfactory physiology through structural, ionic, and neuroprotective mechanisms.

In conclusion, recent insights into melanosome biology, from biogenesis to function, underscore the broader importance of this organelle as an LRO model for the plasticity of the endolysosomal system in various cell types. Melanosomes illustrate how membrane dynamics and cargo transport can be diversified, repurposed, and integrated to support specialized cellular and tissue functions. Extending analyses beyond skin melanocytes to other organisms, cell types, and physiological contexts reveal previously underappreciated roles for melanin, including its contribution to genome photoprotection, stress resilience, and organ-level homeostasis. As comparative and mechanistic studies continue to develop, melanosomes and, overall, pigment organelles, provide a valuable framework for understanding how organelles arise, diverge and interact, and how their dysfunction contributes to disease. These insights highlight the potential of pigmented systems to shed light on fundamental principles of organelle biology and their modulation across evolution, physiology, and pathology.
